# Gummosin, a sesquiterpene coumarin from *Ferula assa-foetida* is preferentially cytotoxic to human breast and prostate cancer cell lines

**Published:** 2019

**Authors:** Milad Iranshahy, Faegheh Farhadi, Babak Paknejad, Parvin Zareian, Mehrdad Iranshahi, Masoumeh Karami, Seyed Reza Abtahi

**Affiliations:** 1 *Department of Pharmacology and Toxicology, Faculty of Medicine, AJA University of Medical Sciences, Tehran, Iran.*; 2 *AJA Cancer Epidemiology Research and Treatment Center (AJA- CERTC), AJA University of Medical Sciences, Tehran, Iran.*; 3 *Department of Pharmacognosy, School of Pharmacy, Mashhad University of Medical Sciences, Mashhad, Iran.*; 4 *Department of Physiology, School of Medicine, AJA University of Medical Sciences, Tehran, Iran.*; 5 *Biotechnology Research Center, Pharmaceutical Technology Institute, Mashhad University of Medical Sciences, Mashhad, Iran.*; 6 *Biochemistry Department, Faculty of Medicine, AJA University of Medical Sciences, Tehran, Iran.*

**Keywords:** Ferula assa-foetida, Apiaceae, Gummosin, Cytotoxicity, MCF-7, PC-3

## Abstract

**Objective:**

The present study was conducted to find cytotoxic compounds from oleo-gum-resin of *Ferula assa-foetida* (asafoetida).

**Materials and Methods:**

A dichloromethane extract of asafoetida was subjected to different chromatography analyses (including column chromatography, preparative thin layer chromatography and high performance liquid chromatography) to isolate its bioactive sesquiterpene coumarins. The structures of isolated compounds were elucidated through ^1^H-NMR spectra interpretation and comparison with those reported in the literature. To measure the cytotoxic activity of pure compounds, a non-fluorescent substrate called resazurin (alamarBlue^®^) was used in this study. Human breast and prostate cancer cell lines (MCF-7 and PC-3, respectively) and a normal human embryonic stem cell (NIH) were treated with different concentrations (50, 25, 12.5 and 6.25 µg/mL) of pure compounds.

**Results:**

In this study, 10 sesquiterpene coumarins were isolated from oleo-gum-resin of *F. assa-foetida* and cytotoxic activity of 6 compounds was tested against MCF-7 and PC-3 cell lines and NIH cells. Badrakemin acetate (7), ferukrinone (8) and deacetyl kellerin (10) were found for the first time in the oleo-gum-resin of *F. assa-foetida*. Gummosin (4) showed moderate cytotoxic activity with IC_50_ values of 30 and 32.1 µg/mL against PC-3 and MCF-7 cell lines, respectively. None of the isolated compounds showed toxicity against NIH as a normal human cell line.

**Conclusion:**

The preferential cytotoxic activity of gummosin against cancer cell lines is reported for the first time in this study.

## Introduction

*Ferula assa-foetida *is an Iranian endemic medicinal plant from the family Apiaceae and its oleo-gum-resin (called asafoetida) has been traditionally used for the treatment of asthma, gastrointestinal disorders, intestinal parasites, nervous disorders and epilepsy (Iranshahy and Iranshahi, 2011 ▶; Akaberi et al., 2015 ▶). However, the most prominent biological characteristics of *Ferula *species are their cytotoxic and anticancer activities (Iranshahi et al., 2018 ▶). Recent studies revealed that asafoetida may have cytotoxic activity against different cancer cell lines including osteosarcoma cell line (HOS CRL), cervical cancer (HeLa) and colorectal cancer (SW620) (Shafri et al., 2015 ▶; Alshammari, 2016 ▶; Bagheri et al., 2017 ▶). The phytochemical composition of asafoetida as an oleo-gum-resin, is very complex and more than 72 compounds were isolated from asafoetida of which, 31 compounds belong to coumarin class of natural compounds. Flavonoids, sulfur-containing compounds and sesquiterpenoids were also found in asafetida (Iranshahy and Iranshahi, 2011 ▶).

 Galbanic acid is the most studied sesquiterpene coumarin owing to its remarkable antitumor and antiangiogenic activities. As galbanic acid is not a potent cytotoxic agent, it seems that its antitumor activity is more likely due to its antiangiogenic activity rather than its direct effects on cancer cells (Kim et al., 2011 ▶; Kasaian et al., 2014 ▶). Farnesiferol C, ferutinin and umbelliprenin are the other coumarins that showed cytotoxicity and could induce apoptosis in human cancer cell lines (Shakeri et al., 2014 ▶; Aldaghi et al., 2016 ▶; Hasanzadeh et al., 2017 ▶; Iranshahi, Rezaee et al., 2018 ▶). Other sesquiterpene coumarins isolated from *Ferula* species were also extensively studied for their cytotoxic activity and in some cases, like ferulenol, the IC_50 _was as low as 1 µg/mL (Iranshahi, 2018 ▶).

Growing lines of evidence show that coumarins are a good scaffold for discovery of anticancer agents because of their structure diversity (kauer et al., 2015 ▶). In addition, sesquiterpene coumarins are potent inhibitors of P-glycoprotein pumps which play a significant role in resistance of cancer cells to chemotherapeutics. Thus, adding a potent sesquiterpene coumarin to current chemotherapy regimens, seems to produce promising results in cancer research (Kasaian et al., 2014 ▶). 

Considering the importance of sesquiterpene coumarins as cytotoxic and anticancer agents, in our ongoing research on biological activities of asafoetida we studied the cytotoxic activity of some sesquiterpene coumarins isolated from asafoetida for the first time. In addition, based on previous studies and our current results, structure-activity relationship (SAR) was also investigated.

## Materials and Methods

**General experimental procedures**

For fractionation and prior to purification using column chromatography (CC) technique, the extract was loaded on silica gel 230–400 mesh (Merck, Germany). The obtained fractions were further purified using a KNAUER semi-preparative HPLC equipped with semi-prep C18 column (Onyx monolithic; 100×10 mm) and a diode-array detector (Smartline DAD 2800). Analytical and preparative TLC were conducted on silica gel 60 F₂₅₄ (Merck, Germany) and silica gel 60 GF₂₅₄ (Merck, Germany), respectively. For structure elucidation, the purified compounds were analyzed using Bruker AVANCE Ш-300 spectrometer (Bruker, Germany).

**Plant materials**

The oleo-gum-resin of *F. assa-foetida* was purchased from the market in April 2017 in Mashhad, Khorasan Razavi Province, Iran. 

**Extraction and isolation**

After grinding, 200 g of the oleo-gum-resin was extracted by dichloromethane (DCM) using maceration in a glass conical flask for 3 days at room temperature. The extract was concentrated under vacuum to give a red extract (80 g); then, 40 g of the extract was subjected to CC over a silica-gel column (230–400 mesh, 100×7 cm). The elution system was started with 100% petroleum ether and 0% ethyl acetate and gradually changed to 0% petroleum ether and 100% ethyl acetate and finally from 100% ethyl acetate to 50% ethyl acetate and 50% methanol to give 39 fractions (Fr.1–39) based on the TLC analysis of the fractions. 

Farnesiferol A (1) from Fr.26, farnesiferol B (2) from Fr.24, and gummosin (4) Fr.25 were extracted and recrystallized using petroleum ether–ethyl acetate (EtOAc) 50:50. Farnesiferol C (3) from Fr.37, samarcandin (5) from Fr.32 and umbelliprenin (6) from Fr.11 were recrystallized using EtOAc – methanol (80:20), EtOAc (100) and petroleum ether–EtOAc (5:1), respectively.

The fraction 20 was subjected to HPLC-C18 column chromatography using aqueous methanol 20% and afforded badrakemin acetate (7). Some fractions that were separated by CC, were further purified by PTLC, after analysis under UV CAMAG spectrometer at 254 nm. Kellerin (9) was obtained from Fr. 28 with DCM - EtOAc (2:0.5) as a solvent system used for multiple runs, while deacetyl kellerin (10) was isolated from fractions 31, using dichloromethane - EtOAc (2:1). 

**Cell culture**

Human breast adenocarcinoma cell line (MCF-7), human prostate cancer cell line (PC3) and human normal fibroblast cell line (NIH) were obtained from Biotechnology Research Center (Mashhad, Iran) and subsequently grown in RPMI-1640 medium with 10% fetal bovine serum (Gibco, Invitrogen, Paisley, UK), penicillin (100 units/ml) and streptomycin (100 mg/ml) as antibiotics. The cell culture was done in a humidified incubator under the atmosphere of 5% CO_2_ at 37˚C.

**AlamarBlue® assay**

To measure the cytotoxic activity of pure compounds, a non-fluorescent substrate called resazurin (alamarBlue^®^; BioSource Invitrogen, Paisley, UK) was used in this study. In this study, we used a method that was previously reported by Haaften et al., with some small modifications (van Haaften et al., 2011). Briefly, when the cells were in the exponential phase, 1×10^4 ^of the three cell lines were seeded in 96-well plates. The cells were incubated for 24 hr in order to adhere to the plastic surface of the bottom of the wells. After 24 hr, the cells were treated with 6.25, 12.5, 25 and 50 µg/mL of each compound from the stock solution of 5 mg/mL in dimethyl sulfoxide (DMSO) via serial solution prepared using RPMI-1640. The amount of DMSO in final culture media was lower than 0.01%. Also, to rule out the effects of DMSO on cells, equal amount of DMSO was added to the untreated groups. After 48 hr, viability of the cells was measured by adding alamarBlue^® ^reagent (10% of tissue culture volume from stock solution of 0.14 mg/mL). The absorbance of the wells was read at 600 nm using a microplate reader. Doxorubicin at concentrations of 0.1, 1 and 5 µg/mL was used as the positive control and the viability was calculated using following formula:

$\mathrm{Viabillity}\%=100-\left[\left(\frac{\left(T-\mathrm{UT}\right)}{\left(B-\mathrm{UT}\right)}\right)\mathrm{*100}\right]$

T=Absorbance of wells with pure compounds at 600 nm after 48 hr of incubation.

UT=Absorbance of wells with cell but without any compound at 600 nm after 48 hr of incubation.

B=Absorbance of blank (without cell and compounds) at 600 nm after 48 hr of incubation.

In this study, all the experiments were done at least in triplicate.

**Statistical analysis**

Data are expressed as mean±SD (standard deviation). For statistical analysis of the data, a one-way analysis of variance (ANOVA) followed by the Dunnett's test was performed by GraphPad Prism 7.04, to compare treated samples with their respective untreated control group and the differences between groups were considered statistically significant when *p* value was lower than 0.05. 

## Results

**Figure 1:**
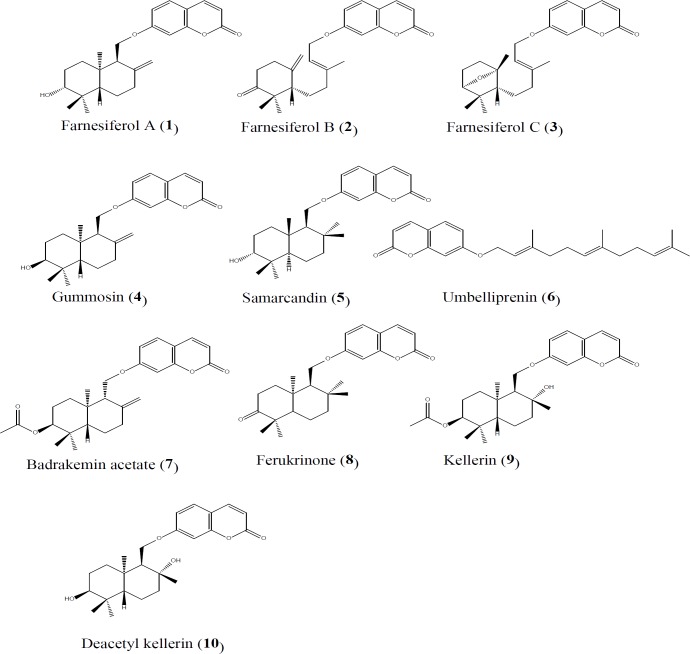
Chemical structures of isolated compounds from *F. assa-foetida*

Ten sesquiterpene coumarins were isolated from asafetida (purity>95%, HPLC) and their chemical structures were confirmed using ^1^H-NMR. The chemical structures of purified compounds including farnesiferol A (1, 1g), farnesiferol B (2, 400 mg), farnesiferol C (3, 240 mg), gummosin (4, 300 mg), samarcandin (5, 500 mg), umbelliprenin (6, 200 mg), badrakemin acetate (**7**, 6 mg), ferukrinone (8, 6 mg), kellerin (9, 5 mg) and deacetyl kellerin (10, 5 mg) are represented in Figure 1. Structure elucidation for the purified compounds was carried out by comparing the ^1^H-NMR spectra of the compounds (S1, supporting information) with those reported in the literature (Iranshahi et al., 2004; Lee et al., 2009; Iranshahi et al., 2010; Kasaian et al., 2015) and interpretation of the ^1^H-NMR spectra. 

The ^1^H NMR spectrum of coumarin nucleus in these compounds was characterized by the doublets (integrating for two protons) at δ 6.24 (1H, d, *J*_˭_ 9.5 Hz), 7.63 (1H, d, *J*_˭_ 9.5 Hz), 7.36 (1H, d, *J*_˭_ 9.2 Hz), and 6.84 (2H, m) (related to *ortho*, *ortho*-*meta* and *meta* couplings of the aromatic protons). In ^13^C NMR spectrum, the signal at 160-162 ppm is assigned to carbonyl group of the lactone ring. To the best of our knowledge, badrakemin acetate, ferukrinone and deacetyl kellerin were not previously isolated from *F. asafoetida* and this is the first report on their isolation. In the ^13^C NMR spectrum of badrakemin acetate, one signal at 170.2 ppm, five signals at 15.5, 18.9, 21, 21 and 21.5 ppm, one signal at 82.3 ppm and one signal at 108 ppm, are indicative of ester carbonyl, methyl, methine and methylene groups and imply the sesquiterpene skeleton. In ^1^H-NMR, the signals appearing at δ 4.03(1H, dd, *J*_˭_ 9.9, 6.3 Hz) and 4.33 (1H, dd, *J*_˭_ 9.9, 5.5 Hz) confirmed the presence of a methylene bridge between the sesquiterpene and O-coumarin residues and a proton resonating at 4.52 ppm (t, *J*=5.9 Hz, 1H, O-C-H) indicates the presence of a methine attached to the acetyl group. ^13^C signals at 218 and 75 ppm, indicative of a carbonyl and a quaternary carbon, and ^1^H signals at 4.21 ppm (dd, *J*=10.6, 2.6 Hz, 1H, -O-CH_2_), 4.13 ppm (dd, *J*=10.6, 3.2 Hz, 1H, -O-CH_2_), δ 2.39 ppm (dd, *J*=5.2, 2.9 Hz, 1H) and 2.34 ppm (dd, *J*=5.2, 2.8 Hz, 1H), indicative of the methylene signals next to a carbonyl group, are characteristic features of ferukrinone. According to ^13^C NMR and ^1^H NMR data, compound 9 differs from ferukinone only in carbon 3. The presence of signals at 21.8, 81.6 and 171.2 ppm and protons resonating at 4.76 ppm (t, *J*=6.9 Hz, 1H, O-C-H) and 2.18 ppm (s, 1H, CH_3_-C=O) are indicative of an acetyl group in carbon 3; thus, compound 9 was identified as kellerin. While these compounds constitute the minor components of the extract (each approximately 5-7 mg), the amount of other six compounds was above 100 mg.

Cytotoxic activity of umbelliprenin, farnesiferol A-C, gummosin and samarcandin was evaluated using alamarBlue^®^ method against PC3, MCF-7 and NIH (normal) cell lines. Farnesiferol B, farnesiferol C and gummosin showed IC_50_ values lower than 50 µg/mL but IC_50_ values for other compounds were above 50 µg/mL (Table 1).

**Table 1:** *In vitro* cytotoxicity of the compounds isolated from oleo-gum-resin of *F. assa-foetida*

No.	Compounds	IC_50_ (µg/mL)±SD/Cell line		
		MCF-7	PC3	NIH
**1**	Farnesiferol A	>50	>50	>100
**2**	Farnesiferol B	42.1±0.79	36.8±2.8	>100
**3**	Farnesiferol C	41.7±8.2	43.1±9.5	>100
**4**	Gummosin	32.1±2.3	30.0±4.7	>100
**5**	Samarcandin	>50	>50	>100
**6**	Umbelliprenin	>50	>50	>100
-	Doxorubicin	0.28	0.62	-

No: Number; SD: standard deviation

## Discussion

**Figure 2:**
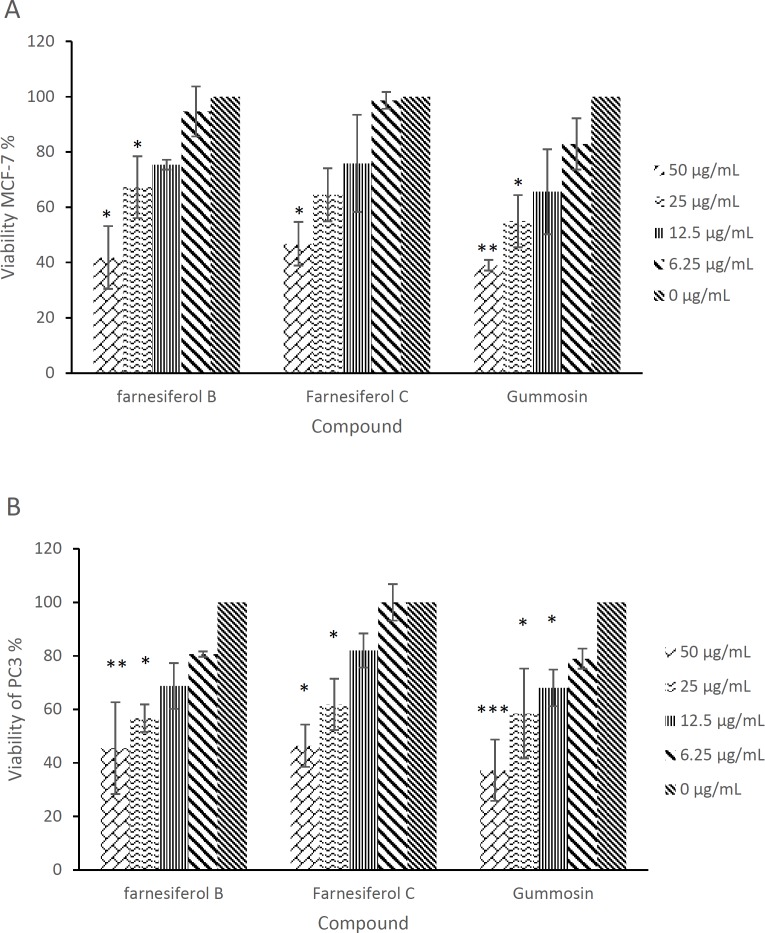
Percentage of MCF-7 (A) and PC3 (B) cancer cells viability following treatment with different concentrations of farnesiferol B-C and gummosin. *, ** and *** indicate *p *values of <0.05, <0.01 and <0.001, respectively when compared with the control group

Evaluating the cytotoxic activity of different concentrations of the compounds tested in the present study showed that the cytotoxicity is dose-dependent and generally, at concentrations <5 µg/mL, the compounds are not able to induce toxicity in the cancer cell lines (Figure 2). Gummosin was the most active compound with IC_50_ values of 32.1 and 30 µg/mL against MCF-7 and PC3 cell lines, respectively. None of the compounds showed cytotoxicity in normal NIH cell line at concentrations up to 100 µg/mL, indicating that these compounds are not cytotoxic for normal cells and are preferentially cytotoxic for cancer cell lines.

Considering the present results and those reported in previous studies, it can be concluded that gummosin is the most cytotoxic sesquiterpene coumarin isolated from *F. assa-foetida* to date. The cytotoxic activity of gummosin seems to be site- and cell-specific since farnesiferol A which has a similar structure to gummosin (the only difference is their stereochemistry of OH group) did not show cytotoxic activity even at its highest dose (50 µg/mL). In addition, gummosin did not cause any toxicity in normal cell line at 50 µg/mL.

Samracandin has a similar structure to gummosin but it lacks a double bond on its sesquiterpene moiety. Samarcandin was unable to induce toxicity even at the highest dose, indicating that the double bond in the sesquiterpene moiety is vital for the cytotoxic activity. Feselol is another sesquiterpene coumarin which has a similar structure to gummosin and they only differ in the presence of a endocyclic double bond instead of an exocyclic one in gummosin. Previous studies showed that this small difference makes feselol inactive against cancer cell lines (Kasaian, et al., 2015 ▶). It can be concluded that beta position of OH and the exocyclic double bond in sesquiterpene moiety are two critical factors required for the cytotoxic activity of sesquiterpene coumarins. Cytotoxic activity of sesquiterpe coumarins isolated from *Ferula *species has been the subject of numerous studies. A bioassay-guided isolation of sesquiterpe coumarins from *F. narthex* led to identification of a new sesquiterpene coumarin with an IC_50_ value of 14 µg/mL against prostate cancer cell line (Alam et al., 2016 ▶). In another study done by our group, cytotoxic activity of 7 sesquiterpene coumarins was evaluated against lung, melanoma, and ovarian cancer cell lines and conferone showed a potent cytotoxic activity against ovarian cancer cell line with an IC_50_ value of 7.79 µM (Valiahdi et al., 2013 ▶).

In conclusion, 10 sesquiterpene coumarins were isolated from *F. assa-foetida* and cytotoxicity of 6 compounds was evaluated against MCF-7 and PC3 cancer cell lines. Badrakemin acetate, ferukrinone and deacetyl kellerin were previously reported from asafoetida. To the best of our knowledge, gummosin was not studied for its cytotoxicity in previous studies and the present study showed a moderate cytotoxicity for gummosin with IC_50 _values of 32.1 and 30 µg/mL against MCF-7 and PC-3, respectively. It seems that beta position of OH and the exocyclic double bond in sesquiterpene moiety are essential for cytotoxicity of sesquiterpene coumarins; also, we found gummosin as the most potent cytotoxic compound isolated from *F. assa-foetida* to date.
